# Domestic

**Published:** 1841-06-05

**Authors:** 


### DOMESTIC.

INTERMENTS *in the City and Liberties of Philadelphia, from the* 22d *to the* 29*th of May,* 1841.

| Diseases. | Adults. | Children. | Diseases. | Adults. | Children |
|---|---|---|---|---|---|
| Apoplexy, | 1 | 0 | *Brought forward,* | 34 | 45 |
| Cancer of womb, | 1 | 0 | Marasmus, | 0 | 2 |
| Casualty, | 0 | 1 | Malformation, | 0 | 1 |
| Croup, | 0 | 1 | Measles, | 1 | 5 |
| Congestion of brain, | 2 | 0 | Mortification of stomach, | 0 | 1 |
| Childbed, | 1 | 0 | Mania a potu, | 1 | 0 |
| Consumption of the lungs, | 12 | 3 | Old age, | 1 | 0 |
| Convulsions, | 1 | 3 | Palsy, | 2 | 0 |
| Dropsy, | 3 | 1 | Pleuropneumonia, | 3 | 0 |
| —— head, | 0 | 4 | Puerperal mania, | 1 | 0 |
| —— breast, | 1 | 0 | Scrofula, | 0 | 4 |
| Disease of brain, | 0 | 1 | Small pox, | 1 | 3 |
| —— spine, | 0 | 1 | Still-born, | 0 | 8 |
| —— liver, | 1 | 0 | Suicide, | 1 | 0 |
| —— stomach, | 1 | 0 | Unknown, | 1 | 1 |
| Drowned, | 0 | 3 | Total, 116— | 46 | 70 |
| Dysentery, | 1 | 1 | | | |
| Debility, | 0 | 3 | | | |
| Erysipelas, | 0 | 2 | | | |
| Enlargement of liver, | 0 | 1 | | | |
| Fever, | 1 | 0 | | | |
| —— remittent, | 0 | 1 | | | |
| —— nervous | 1 | 0 | | | |
| Inflammation of the brain, | 1 | 6 | | | |
| —— bronchi, | 1 | 2 | | | |
| —— lungs, | 1 | 5 | | | |
| —— stomach, | 1 | 0 | | | |
| —— bowels, | 1 | 3 | | | |
| —— larynx, | 0 | 1 | | | |
| —— breast, | 0 | 1 | | | |
| —— peritonæum, | 2 | 0 | | | |
| Inanition, | 0 | 1 | | | |
| *Carried forward,* | 34 | 45 | | | |

Of the above, there were under 1 year 33

| | |
|---|---|
| From 1 to 2 | 13 |
| 2 to 5 | 7 |
| 5 to 10 | 8 |
| 10 to 15 | 3 |
| 15 to 20 | 6 |
| 20 to 30 | 15 |
| 30 to 40 | 12 |
| 40 to 50 | 7 |
| 50 to 60 | 7 |
| 60 to 70 | 1 |
| 70 to 80 | 4 |
| 80 to 90 | 0 |
| 90 to 100 | 0 |
| Total, | 116 |

Of the above there were 8 from the almshouse, 20 people of colour, and one from the country, which are included in the total amount.

